# A Novel Nomogram Model to Identify Candidates and Predict the Possibility of Benefit From Primary Tumor Resection Among Female Patients With Metastatic Infiltrating Duct Carcinoma of the Breast: A Large Cohort Study

**DOI:** 10.3389/fonc.2022.798016

**Published:** 2022-02-14

**Authors:** Ziqiong Wang, Bo Chen, Jiyang Chen, Zhixuan Wu, Hongyi Gu, Ying Wang, Xuanxuan Dai

**Affiliations:** ^1^ Department of Thyroid and Breast Surgery, The First Affiliated Hospital of Wenzhou Medical University, Wenzhou, China; ^2^ The First Clinical College, Wenzhou Medical University, Wenzhou, China; ^3^ Department of Hepatobiliary Surgery, The First Affiliated Hospital of Wenzhou Medical University, Wenzhou, China

**Keywords:** stage IV breast cancer, propensity score matching, surgery, prognosis, nomogram

## Abstract

**Background:**

The impact of primary site surgery on survival remains controversial in female patients with stage IV breast cancer. The purpose of this study was to investigate the role of primary tumor surgery in patients with stage IV breast cancer and concurrently develop a nomogram to identify which patients will benefit from surgery.

**Methods:**

We retrospectively searched the SEER database for female patients newly diagnosed with stage IV breast infiltrating duct carcinoma (BIDC) between 2010 and 2015 and then divided them into surgery and non-surgery groups. The propensity score matching (PSM) method was implemented to eliminate the bias, and Kaplan–Meier survival analysis was generated to compare the overall survival (OS) and cancer-specific survival (CSS) between the two groups. After PSM, Cox regression analyses were performed to determine the independent protective value of primary tumor surgery, while logistic regression analyses were utilized to uncover significant predictors of surgical benefit and establish a screening nomogram for female patients with stage IV BIDC. Nomogram performance was evaluated by calibration curves, receiver operating characteristic (ROC) curves, and decision curve analysis (DCA).

**Result:**

5,475 patients with stage IV BIDC were included in this study, and 2,375 patients (43.38%) received primary tumor surgery. After PSM, the median CSS was 53 months (95% CI: 46.84–59.16) in the surgery group compared with only 33 months (95% CI: 30.05–35.95) in the non-surgery group. We further found that primary tumor surgery was an independent protective factor for patients with stage IV BIDC. The independent factors affecting the benefit of locoregional surgery in patients with stage IV BIDC included histological grade, T stage, molecular subtype, lung metastasis, liver metastasis, brain metastasis, and marital status. The AUC of the nomogram was 0.785 in the training set and 0.761 in the testing set. The calibration curves and DCA confirmed that the nomogram could precisely predict the possibility of benefit from primary tumor resection.

**Conclusion:**

Our study suggested that primary tumor surgery improved the prognosis of female patients with stage IV BIDC and developed a nomogram to quantify the probability of surgical benefit to help identify surgical candidates clinically.

## Introduction

Breast cancer (BC) is a common malignant tumor and the second most common cause of cancer death among women in the United States (1). Owing to the intense effort in public education and effective screening, approximately 90% of BC patients present early-stage disease at the time of diagnosis (2). Early-stage BC is considered as a curable disease with standard surgical resection and radiation, with a 5-year survival rate of over 90% (3, 4). Although early-stage BC presents an excellent prognosis, it is virtually incurable once tumor cells spread to distant sites (5). Additionally, approximately 25%–30% of early-stage BC metastasizes and progresses to advanced BC, which is the leading cause of death from BC (6), and only 24%–26.5% of patients with stage IV BC survive for more than 5 years (7, 8).

Systemic therapy is the main treatment for stage IV BC patients to relieve symptoms, improve quality of life, and prolong survival, including endocrine therapy, targeted drug therapy, chemotherapy, and radiotherapy (9, 10). Moreover, amplitude-modulated radiofrequency electromagnetic fields (AM RF EMF) at breast cancer-specific frequencies can result in complete and partial responses in patients with stage IV BC (11). However, the efficacy of surgical resection of the primary site in patients with stage IV BC remains controversial. Metastatic BC represents a major clinical problem as it is hard to be surgically resected, unlike the primary tumor (12). In clinical practice, primary tumor resection is not a routine treatment for patients with stage IV BC, but only to relieve chest symptoms such as bleeding, ulcers, and pain due to chest wall invasion (13). Several studies indicated that surgical intervention not only failed to improve the survival rate of patients with metastatic BC but also created a permissive environment for tumor relapses and distant metastases (14). The possible reason for this phenomenon may be that the surgery causes cancer cells to enter the circulation (15). Yu et al. combined prospective clinical multicenter trials and found that locoregional surgery did not prolong overall survival (OS) of stage IV BC patients but had a significantly longer locoregional progression-free survival (PFS) (16). Similarly, the ECOG-E2018 study reported the results of a randomized phase III trial that showed no significant difference in OS or PFS in patients with stage IV BC who received systemic therapy plus early local therapy versus systemic therapy alone. Conversely, Khan et al. and Thomas et al. generated two retrospective population-based studies and the results indicated that local tumor resection had a positive impact on survival in patients with stage IV BC (17, 18). Therefore, it is of great importance to clarify the effect of primary tumor resection on the survival of female patients with stage IV BC and develop a novel model to quantify the probability of surgical benefit to help identify surgical candidates clinically.

It is well known that the histological subtype of BC can affect prognosis, with approximately 70%–80% of BCs being infiltrating duct carcinoma (IDC) (19), so we selected female patients with stage IV breast IDC (BIDC) as research objects. Thus, we identified a large representative stage IV BIDC cohort to evaluate the impact of primary site surgery on survival after propensity score matching (PSM) and explore the independent protective value of locoregional surgery. Then, we established a nomogram to identify candidates and quantify the probability of surgical benefit in female patients with stage IV BIDC.

## Methods

### Population Selection

The data included in this study were obtained from the Surveillance, Epidemiology, and End Results (SEER) database. The analysis of unidentified data in the SEER database did not require informed consent and was exempt from medical ethics review. We retrospectively searched for the data of female patients with stage IV BIDC from 2010 to 2015 and conducted a retrospective study. Patients who met the following inclusion criteria were included into the research: (1) 20 ≤ age ≤80; (2) first tumor; (3) histologically diagnosed as IDC; (4) survival time ≥1 month; (5) demographic variables and tumor characteristics are all available. In addition, patients diagnosed with autopsy or death certificate were excluded from this study. Totally, 5,475 patients (2,375 in the surgery group and 3,100 patients in the non-surgery group) were included to form a PSM cohort to explore the impact of locoregional surgery on survival in female patients with stage IV BIDC. Afterward, 2,375 patients in the surgery group were randomly divided into the training group and testing group in a ratio of 7:3. Patients in the training set were used to develop the model, and patients in the testing set were used to validate the performance of the model.

### Statistical Analysis

Statistical analysis was conducted using SPSS 24.0 and R 4.0.2. All statistical tests were bilateral, and p < 0.05 was considered statistically significant. After stage IV BIDC patients were divided into surgery and non-surgery groups, the PSM method was implemented to construct paired matched samples of two treatment groups to balance confounding variables. More specifically, patients in the two groups were 1:1 matched on the logit scale, using variables of p < 0.05 to generate propensity scores, with a caliper value of 0.0001. Then, the differences of variables between the surgery and non-surgery groups (before and after PSM) were evaluated by the chi-square test. We determined overall survival (OS) and cancer-specific survival (CSS) as two primary endpoints in this research. OS was measured as the time from BC diagnosis to the date of death due to any cause (including BC) or the date of last follow-up. CSS was measured as the time from the date of BC diagnosis to the date of BC death or the date of last follow-up. Subsequently, the Kaplan–Meier (K-M) method with the log-rank test was generated to observe the differences in OS and CSS between the surgery and non-surgery groups. Additionally, univariate and multivariate Cox regression analyses were performed to assess the independent protective value of primary tumor resection, and to identify CSS-related independent clinicopathological factors.

### Construction and Validation of a Screening Nomogram to Identify Candidates With Stage IV BIDC for Primary Tumor Resection

We hypothesized that not all female patients with stage IV BIDC would benefit from primary tumor surgery. Under this assumption, patients who received primary tumor resection were divided into two groups, the benefit group and the non-benefit group, based on the median CSS time of the non-surgery group after PSM (33 months). Then, the univariate logistic analysis was used to determine the factors affecting the benefit of locoregional surgery. Significant variables with p < 0.05 in the univariate analysis were incorporated into the multivariate logistic analysis to further reveal the independent predictors of surgical benefit for patients with stage IV BIDC. Subsequently, on the basis of the surgery-benefit-associated factors, we established a novel nomogram with the “rms” package, a simple, multivariate oncology visualization tool for predicting and quantifying outcome rates for individual patients (20), to identify candidates with stage IV BIDC for primary tumor resection and quantify the probability of surgical benefit. To validate the performance of the screening nomogram for patients with stage IV BIDC, we generated the receiver operating characteristic (ROC) curves and compared the corresponding area under the curve (AUC) values of the nomogram and individual surgery-benefit-associated factor in the training and testing sets, respectively. Furthermore, the discrimination and clinical practicability of the nomogram were evaluated by the calibration plot and decision curve analysis (DCA).

Moreover, to further verify the applicability of the model in the absence of prospective research data, we assessed the performance of the nomogram in the PSM cohort. Based on the score of each patient calculated by the nomogram, we divided all female patients with stage IV BIDC into three groups: (1) Surgery-Benefit group; (2) Surgery-Nonbenefit group; and (3) Non-surgery group. Specifically, patients in the surgery group with a probability (calculated by the nomogram score) greater than 50% were assigned to the Surgery-Benefit group, while others were assigned to the Surgery-Nonbenefit group. Finally, K-M survival analysis with the log-rank test (overall and pairwise comparisons) was implemented to compare the CSS status among the above three groups, so as to test whether the model could quantify the probability of surgical benefit and identify surgical candidates.

## Results

### Clinicopathological Characteristics of the Patients With Stage IV BIDC

**Figure 1:**
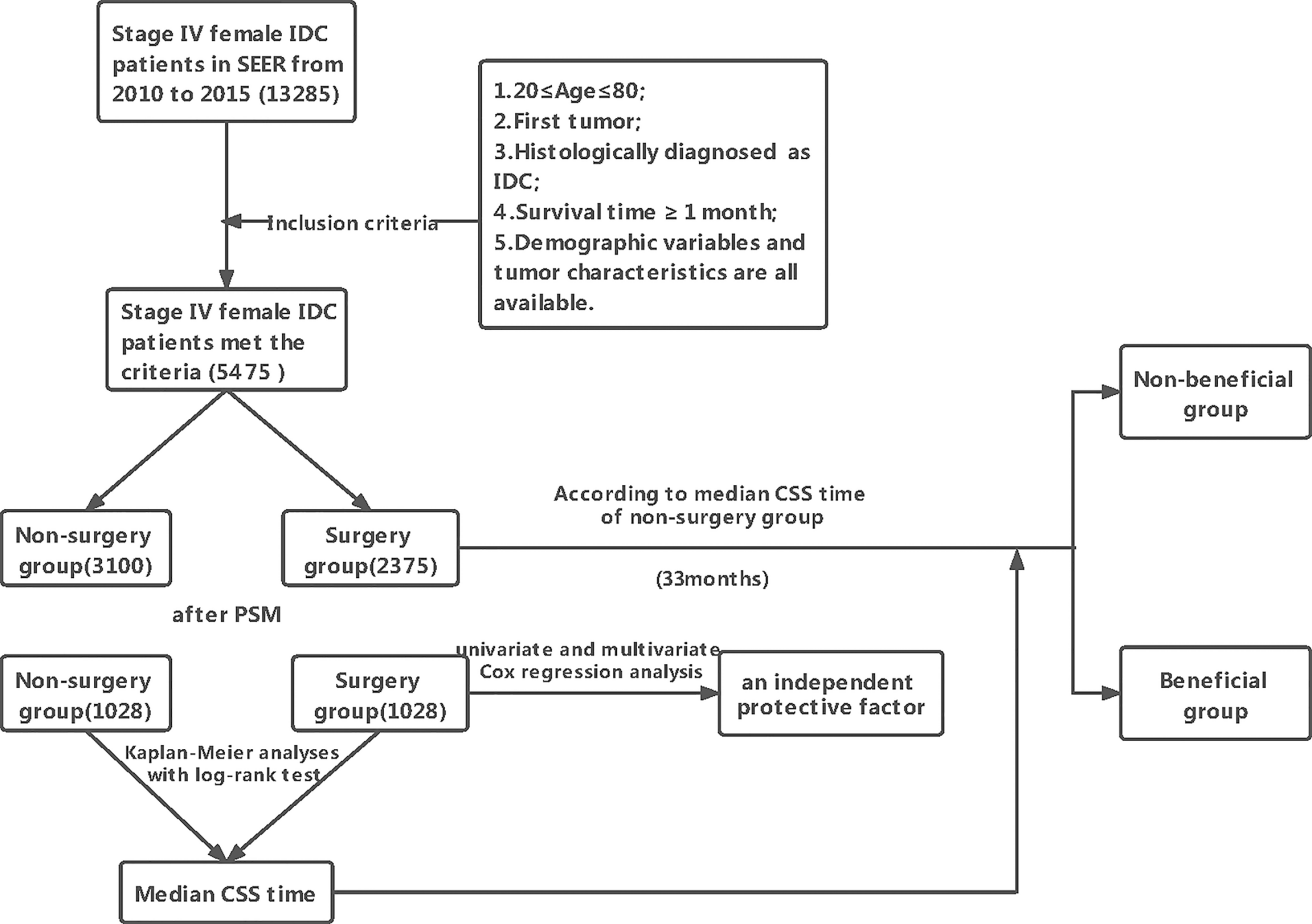
General flowchart of this study.

In total, 13,285 female patients with stage IV BIDC at initial diagnosis between 2010 and 2015 were included in this study and 5,475 met the criteria **(** **Figure 1** **)**. As shown in **Table 1**, 3,100 patients (56.62%) did not receive primary tumor surgery and 2,375 did (43.38%). There were significant differences between these two groups. Patients with locoregional surgery were more likely to be younger, married, and insured, and have higher histologic grade, higher T stage, higher N stage, and higher proportion of bone-only metastasis. Furthermore, those who received chemotherapy and radiation therapy also tended to undergo locoregional surgery. This indicated an imbalance in the baseline characteristics between the two groups.

**Table 1:** Clinical and pathological characteristics for female patients with stage IV IDC of breast before PSM.

	Surgery group (n = 2,375)	Non-Surgery group (n = 3,100)	X^2^	p
Age, years			32.421	0.000
≤40	333	310		
41–60	1,172	1,464		
≥61	870	1,326		
Race			0.260	0.878
Black	430	565		
Other	217	271		
White	1,728	2,264		
Primary site			9.304	0.098
Central portion	152	198		
Upper inner	198	239		
Lower inner	120	133		
Upper outer	700	848		
Lower outer	162	199		
Others	1,043	1,483		
Laterality			0.157	0.692
Left	1,213	1,600		
Right	1,162	1,500		
Grade			99.915	0.000
I	113	186		
II	734	1,337		
III+IV	1,528	1,577		
T			29.193	0.000
T1	286	392		
T2	981	1,066		
T3	429	606		
T4	679	1,036		
N			48.822	0.000
N0	369	701		
N1–3	2,006	2,399		
Radiotherapy			215.644	0.000
No	1,168	2,132		
Yes	1,207	968		
Chemotherapy			134.105	0.000
No	549	1,171		
Yes	1,826	1,929		
Bone metastasis			110.936	0.000
No	1,059	953		
Yes	1,316	2,147		
Brain metastasis			53.067	0.000
No	2,284	2,828		
Yes	91	272		
Liver metastasis			53.194	0.000
No	1,853	2,145		
Yes	522	955		
Lung metastasis			92.958	0.000
No	1,804	1,978		
Yes	571	1,122		
Breast subtype			37.396	0.000
HR-/HER2-	447	418		
HR-/HER2+	269	302		
HR+/HER2-	1,199	1,757		
HR+/HER2+	460	623		
Tumor size			5.941	0.051
≤20	331	483		
21–50	1,174	1,436		
≥51	870	1,181		
Insurance status			14.120	0.000
Uninsured	71	156		
Insured	2,304	2,944		
Marital status			20.420	0.000
Married	1,258	1,460		
Discovered	349	474		
Single	534	821		
Widowed	234	345		

IDC, infiltrating duct carcinoma; PSM, propensity score matching.

To eliminate the patient-dependent bias, the 1:1 matched PSM method was generated, and 2,056 female patients with stage IV BIDC (1,028 in the surgery group and 1,028 in the non-surgery group) were included for the following survival analysis. Baseline characteristics, including age, race, primary site, laterality, histologic grade, T stage, N stage, radiation therapy, chemotherapy, metastatic site (bone, brain, liver, lung), molecular subtype, tumor size, insurance status, and marital status, were all balanced (p > 0.05), as seen in **Table 2**. Moreover, we compared the characteristics for the surgery/non-surgery group before and after PSM **(** **Supplementary Tables 1**, **2** **)**. The distributions of grade, N stage, radiotherapy status, chemotherapy status, bone metastasis status, liver metastasis, and molecular subtype were unbalanced for patients in the surgery group before and after PSM, while grade, T stage, N stage, radiotherapy status, chemotherapy status, bone metastasis status, brain metastasis status, liver metastasis status, lung metastasis status, and insurance status were unevenly distributed for the non-surgery group.

**Table 2:** Clinical and pathological characteristics for female patients with stage IV IDC of breast after PSM.

	Surgery group (n = 1,028)	Non-Surgery group (n = 1,028)	X^2^	p
Age, years			0.474	0.789
≤40	126	116		
41–60	485	492		
≥61	417	420		
Race			0.186	0.911
Black	173	171		
Other	90	85		
White	765	772		
Primary site			3.241	0.663
Central portion	75	67		
Upper inner	75	81		
Lower inner	52	39		
Upper outer	298	311		
Lower outer	58	65		
Others	470	465		
Laterality			0.049	0.825
Left	532	527		
Right	496	501		
Grade			4.306	0.116
I	59	40		
II	398	421		
III+IV	571	567		
T			4.299	0.231
T1	130	130		
T2	414	392		
T3	174	210		
T4	310	296		
N			0.028	0.867
N0	196	199		
N1–3	832	829		
Radiotherapy			0.862	0.353
No	684	664		
Yes	344	364		
Chemotherapy			0.056	0.812
No	321	326		
Yes	707	702		
Bone metastasis			0.019	0.890
No	368	365		
Yes	660	663		
Brain metastasis			0.000	1
No	977	977		
Yes	51	51		
Liver metastasis			0.367	0.545
No	770	758		
Yes	258	270		
Lung metastasis			0.421	0.516
No	761	748		
Yes	267	280		
Breast subtype			3.627	0.305
HR-/HER2-	141	154		
HR-/HER2+	112	93		
HR+/HER2-	561	585		
HR+/HER2+	214	196		
Tumor size			0.283	0.868
≤20	155	155		
21–50	504	493		
≥51	369	380		
Insurance status			1.032	0.310
Uninsured	28	36		
Insured	1,000	992		
Marital status			3.095	0.377
Married	538	517		
Discovered	135	147		
Single	235	259		
Widowed	120	105		

IDC, infiltrating duct carcinoma; PSM, propensity score matching.

### The Impart of Primary Tumor Resection on the Survival Outcomes of Patients With Stage IV BIDC

**Figure 2:**
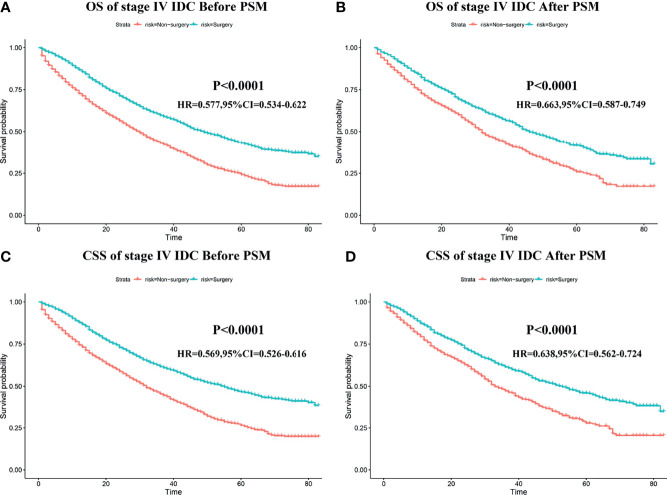
The impart of primary tumor resection on the survival outcomes of patients with stage IV BIDC. Kaplan–Meier (K-M) survival curves of overall survival (OS) before PSM **(A)** and after PSM **(B)**, and cancer-specific survival (CSS) before PSM **(C)** and after PSM **(D)** in the surgery and non-surgery groups.

The median OS time, median CSS time, and 1–5-year OS/CSS survival rate for the surgery and non-surgery groups (before and after PSM) are shown in **Supplementary Table 3**. After eliminating the patient-dependent bias of the surgery and non-surgery groups with the help of PSM analysis, the effect of primary tumor resection on the prognosis of patients with stage IV BIDC could be further studied. As shown in **Figures 2A–D**, the K-M survival analysis indicated that, among patients in the 1:1 matched group, those who underwent primary tumor resection had longer OS and CSS time both before and after PSM than those who did not. The median CSS time was 53 months (95% CI: 46.84–59.16) in the surgery group as compared to only 33 months (95% CI: 30.05–35.95) in the non-surgery group (**Table 3**). Additionally, the 3-year CSS rate and 5-year CSS rate were 0.641 (95% CI: 0.583–0.648) and 0.459 (95% CI: 0.422–0.501) for the surgery group and 0.473 (95% CI: 0.439–0.510) and 0.281 (95% CI: 0.240-0.330) for the non-surgery group **(** **Table 4** **)**. To further investigate the prognostic role of locoregional surgery, univariate and multivariate analyses for CSS were implemented, and the result concluded that primary tumor resection was clearly an independent protective factor **(** **Table 5** **)**. Other than that, age, histological grade, T stage, site of metastasis (bone, brain, liver, lung), molecular subtype, and marital status could also independently affect the clinical outcome of patients with stage IV BIDC **(** **Table 5** **)**.

**Table 3:** Comparison of median survival time between the two groups of patients.

	Before PSM		After PSM	
	Surgery vs. non-Surgery (HR; 95%CI)	p value	Surgery vs. non-Surgery (HR; 95%CI)	p value
Median OS	49 vs. 30 (0.577; 0.534–0.622)	<0.001	46 vs. 32 (0.663; 0.587–0.749)	<0.001
Median CSS	55 vs. 32 (0.569; 0.526–0.616)	<0.001	53 vs. 33 (0.638; 0.562–0.724)	<0.001

PSM, propensity score match; HR, hazard rate; OS, overall survival; CSS, cancer-specific survival; CI, confidence interval.

**Table 4:** Comparison of patient survival rates between the two groups in the PSM cohort.

	OS	CSS
	Surgery vs. Non-Surgery (95% CI)	Surgery vs. Non-Surgery (95% CI)
3-year survival rate	0.592 (0.560–0.625) vs. 0.454 (0.421–0.490)	0.614 (0.583–0.648) vs. 0.473 (0.439–0.510)
5-year survival rate	0.416 (0.379–0.456) vs. 0.258 (0.218–0.305)	0.459 (0.422-0.501) vs. 0.281 (0.240–0.330)

OS, overall survival; CSS, cancer-specific survival.

**Table 5:** Univariate and multivariate Cox analyses for CSS among PSM population.

	Univariate analysis			Multivariate analysis		
	HR	95%CI	P	HR	95%CI	P
Age, years						
≤40	Reference		0.002	Reference		0.033
41-60	1.486	1.190-1.854	0.000	1.349	1.077-1.691	0.009
≥61	1.382	1.102-1.732	0.005	1.271	1.002-1.611	0.048
Race						
Black	Reference		0.000			
Other	0.711	0.549-0.920	0.010			
White	0.673	0.574-0.789	0.000			
Laterality						
Left	Reference					
Right	0.951	0.839-1.078	0.430			
Primary site						
Central portion	Reference		0.406			
Upper inner	0.980	0.714-1.346	0.903			
Lower inner	0.808	0.551-1.185	0.275			
Upper outer	0.947	0.736-1.218	0.672			
Lower outer	0.705	0.490-1.014	0.060			
Others	0.919	0.720-1.172	0.495			
Grade						
I	Reference		0.000	Reference		0.000
II	1.834	1.221-2.755	0.003	1.723	1.143-2.596	0.009
III+IV	3.258	2.183-4.862	0.000	2.511	1.665-3.788	0.000
T						
T1	Reference		0.000	Reference		0.000
T2	1.125	0.902-1.404	0.297	1.097	0.877-1.371	0.418
T3	1.435	1.127-1.826	0.003	1.174	0.919-1.500	0.198
T4	1.745	1.397-2.180	0.000	1.494	1.190-1.876	0.001
N						
N0	Reference					
N1-3	1.111	0.944-1.307	0.205			
Surgery						
No	Reference			Reference		
Yes	0.638	0.562-0.724	0.000	0.648	0.570-0.736	0.000
Radiotherapy						
No	Reference					
Yes	1.098	0.964-1.251	0.160			
Chemotherapy						
No	Reference					
Yes	1.031	0.901-1.180	0.657			
Bone metastasis						
No	Reference			Reference		
Yes	0.825	0.725-0.939	0.003	1.162	1.009-1.339	0.037
Brain metastasis						
No	Reference			Reference		
Yes	2.492	1.984-3.129	0.000	2.073	1.641-2.619	0.000
Liver metastasis						
No	Reference			Reference		
Yes	1.467	1.279-1.683	0.000	1.541	1.333-1.781	0.000
Lung metastasis						
No	Reference			Reference		
Yes	1.416	1.236-1.622	0.000	1.226	1.059-1.419	0.006
Breast subtype						
HR-/HER2-	Reference		0.000	Reference		0.000
HR-/HER2+	0.272	0.211-0.350	0.000	0.276	0.213-0.357	0.000
HR+/HER2-	0.293	0.250-0.344	0.000	0.383	0.321-0.456	0.000
HR+/HER2+	0.203	0.164-0.252	0.000	0.223	0.179-0.278	0.000
Tumor size						
≤20	Reference		0.000			
21-50	1.101	0.907-1.337	0.331			
≥51	1.473	1.208-1.795	0.000			
Insurance status						
Uninsured	Reference					
Insured	0.666	0.481-0.921	0.014			
Marital status						
Married	Reference		0.000	Reference		0.000
Discovered	1.457	1.211-1.754	0.000	1.328	1.100-1.603	0.003
Single	1.420	1.219-1.655	0.000	1.327	1.136-1.549	0.000
Widowed	1.606	1.316-1.960	0.000	1.603	1.303-1.972	0.000

OS, overall survival; PSM, propensity score matching; HR, hazard radio; CI, confidence interval.

### A Nomogram to Screen Candidates With Stage IV BIDC for Primary Tumor Resection

**Figure 3:**
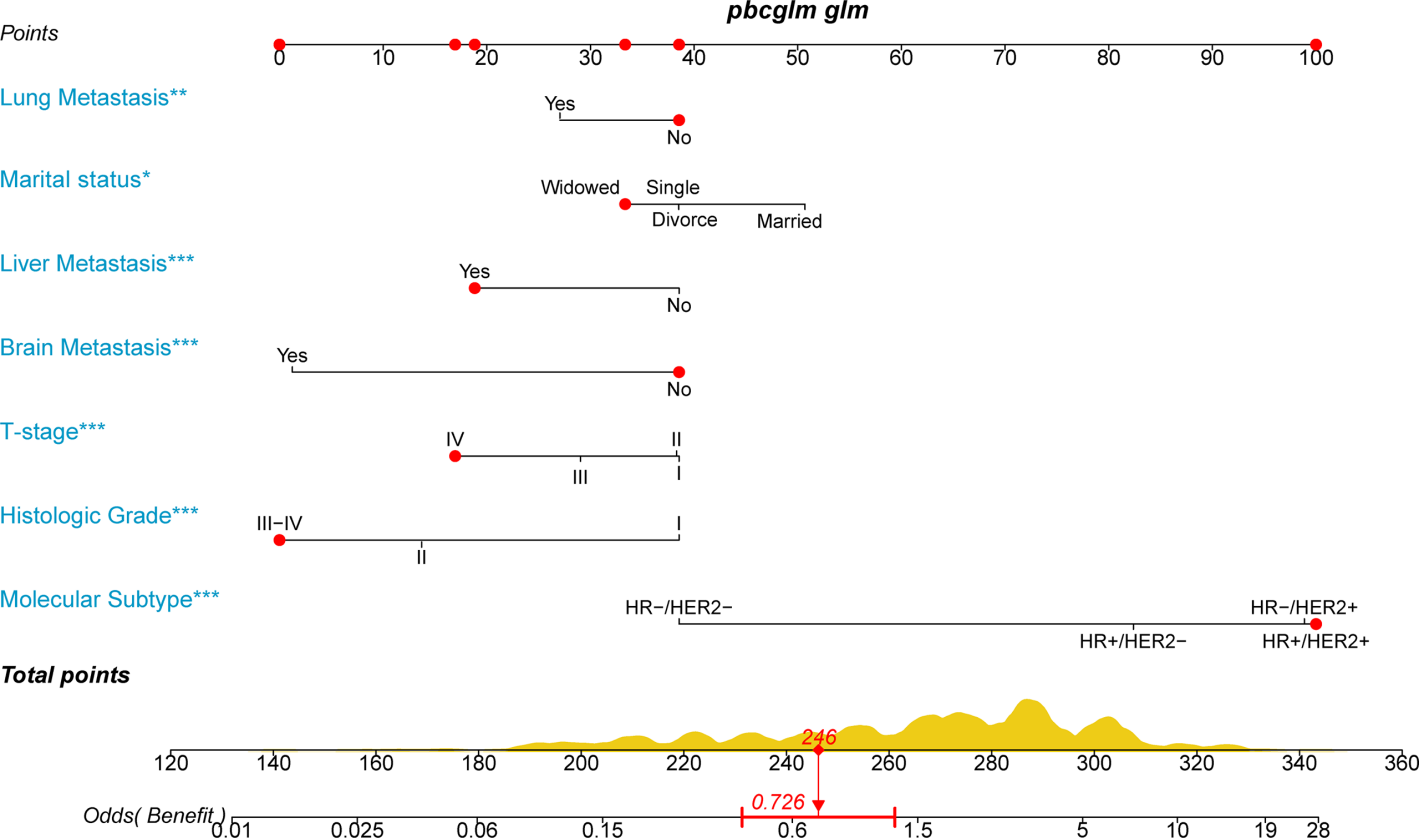
A novel nomogram to select candidates with stage IV BIDC for primary site surgery and predict the possibility of surgical benefit.

We hypothesized that not all patients would benefit from primary site surgery and defined that only patients in the surgery group who had a median CSS time longer than the non-surgery group (33 months) would benefit. For the rigor of this study, we deleted two types of patients (n = 563) in the surgery group who could not be determined as benefit or not, as follows: (1) CSS status = 0 and survival time < 33 months and (2) CSS status = 1 and survival time = 33 months. With this assumption, patients in the surgery group with a median CSS time of more than 33 months were categorized as the benefit group (1,064 patients), while those with less than 33 months were categorized as the non-benefit group (748 patients). Thereafter, univariate and multivariate logistic analyses were performed to determine the independent factors influencing the benefit from primary tumor resection of patients with stage IV BIDC. The independent surgery-benefit-associated predictors for primary tumor resection included histologic grade (p < 0.001), T stage (p < 0.001), molecular subtype (p < 0.001), lung metastasis (p = 0.005), liver metastasis (p < 0.001), brain metastasis (p < 0.001), and marital status (p = 0.002) **(** **Table 6** **)**. Based on the above seven independent factors, a novel nomogram was established to identify female candidates with stage IV BIDC for locoregional surgery (**Figure 3**). By adding up the score for each corresponding variable, the resulting total score could quantify the probability of surgical benefit.

**Table 6:** Univariate and multivariate logistic analyses of benefit in female patients with stage IV IDC of breast.

	Univariate analysis				Multivariate analysis		
	OR	95% CI		p	OR	95% CI	p
Age, years							
≤40	Reference			0.002			
41–60	0.528	0.368–0.758		0.001			
≥61	0.535	0.368–0.778		0.001			
Race							
Black	Reference			0.001			
Other	1.892	1.194–2.999		0.007			
White	1.742	1.304–2.329		0.000			
Laterality							
Left	Reference						
Right	0.948	0.758–1.185		0.638			
Primary site							
Central portion	Reference			0.409			
Upper inner	0.981	0.545–1.766		0.948			
Lower inner	1.012	0.518–1.975		0.973			
Upper outer	0.796	0.492–1.289		0.354			
Lower outer	1.349	0.704–2.585		0.367			
Others	0.843	0.530–1.342		0.471			
Grade							
I	Reference			0.000	Reference		0.000
II	0.421	0.200–0.882		0.022	0.394	0.177–0.876	0.022
III+IV	0.172	0.084–0.352		0.000	0.236	0.108–0.516	0.000
T							
T1	Reference			0.000	Reference		0.000
T2	0.813	0.558–1.184		0.280	0.991	0.647–1.518	0.967
T3	0.612	0.401–0.934		0.023	0.700	0.434–1.130	0.144
T4	0.329	0.224–0.484		0.000	0.445	0.286–0.691	0.000
N							
N0	Reference						
N1–3	0.888	0.658–1.199		0.440			
Bone metastasis							
No	Reference						
Yes	1.244	0.993–1.559		0.057			
Brain metastasis							
No	Reference				Reference		
Yes	0.275	0.161–0.470		0.000	0.247	0.135–0.452	0.000
Liver metastasis							
No	Reference				Reference		
Yes	0.571	0.439–0.743		0.000	0.478	0.351–0.650	0.000
Lung metastasis							
No	Reference				Reference		
Yes	0.503	0.389–0.651		0.000	0.650	0.480–0.880	0.005
Breast subtype							
HR-/HER2-	Reference			0.000	Reference		0.000
HR-/HER2+	8.145	5.096–13.020		0.000	9.595	5.821–15.818	0.000
HR+/HER2-	7.098	5.059–9.958		0.000	5.170	3.584–7.458	0.000
HR+/HER2+	10.461	6.940–15.768		0.000	10.008	6.472–15.477	0.000
Tumor size							
≤20	Reference			0.000			
21–50	0.875	0.626–1.224		0.436			
≥51	0.476	0.337–0.671		0.000			
Insurance status							
Uninsured	Reference						
Insured	1.593	0.834–3.042		0.158			
Marital status							
Married	Reference			0.000	Reference		0.002
Discovered	0.635	0.455–0.885		0.007	0.634	0.431–0.933	0.021
Single	0.643	0.486–0.850		0.002	0.634	0.459–0.874	0.005
Widowed	0.506	0.351–0.730		0.000	0.522	0.342–0.796	0.003

IDC, infiltrating duct carcinoma; OR, odds radio; CI, confidence interval.

### Validation of the Screening Nomogram and the Assumption

**Figure 4:**
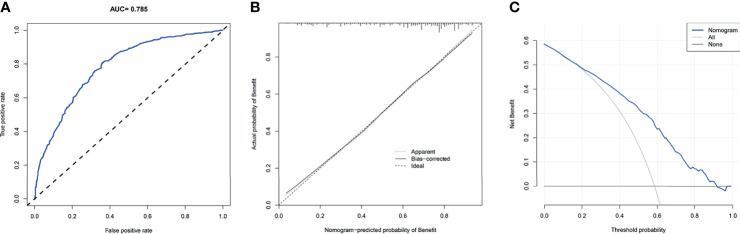
The receiver operating characteristic curve **(A)**, calibration curve **(B)**, and decision curve analysis **(C)** of the screening nomogram in the training set.

**Figure 5:**
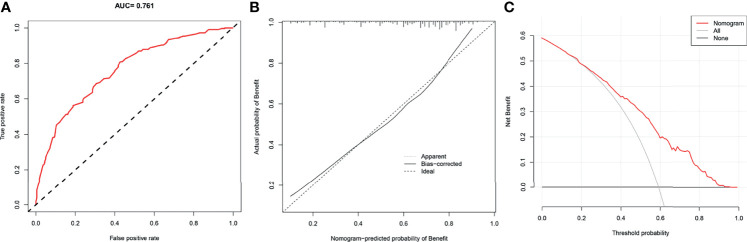
The receiver operating characteristic curve **(A)**, calibration curve **(B)**, and decision curve analysis **(C)** of the screening nomogram in the testing set.

**Figure 6:**
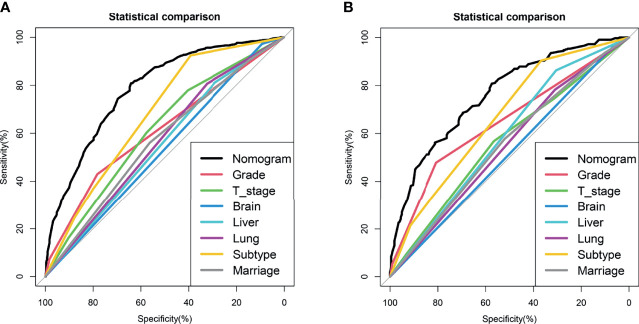
Comparison of area under the receiver operating characteristic curve between nomogram and each independent predictors in the training set **(A)** and the testing set **(B)**.

We drew ROC curves for the training and testing sets, with the AUC of the nomogram being 0.785 in the training set and 0.761 in the testing set **(** **Figure 4A** and **Figure 5A** **)**. As shown in **Figure 4B** and **Figure 5B**, the calibration curves showed a good correlation between predictions and actual observations for patients with stage IV BIDC both in the training and testing sets. Besides, DCA curves also suggested that the nomogram presented good predictive ability and could be a precise tool for identifying surgical candidates clinically **(** **Figure 4C** and **Figure 5C** **)**. In addition, ROC curves were also generated for each independent predictor variable **(** **Figures 6A, B** **)**, and the results implied that the AUC of the nomogram was higher than the AUC of all predictors individually, in both the training and testing sets. Additionally, we further validated the screening nomogram in the entire cohort and obtained good results with an AUC of 0.778 **(** **Supplementary Figure 1A** **)**. The calibration plot also presented good agreement, and DCA showed the prediction accuracy in a wider range **(** **Supplementary Figures 1B, C** **)**.

**Figure 7:**
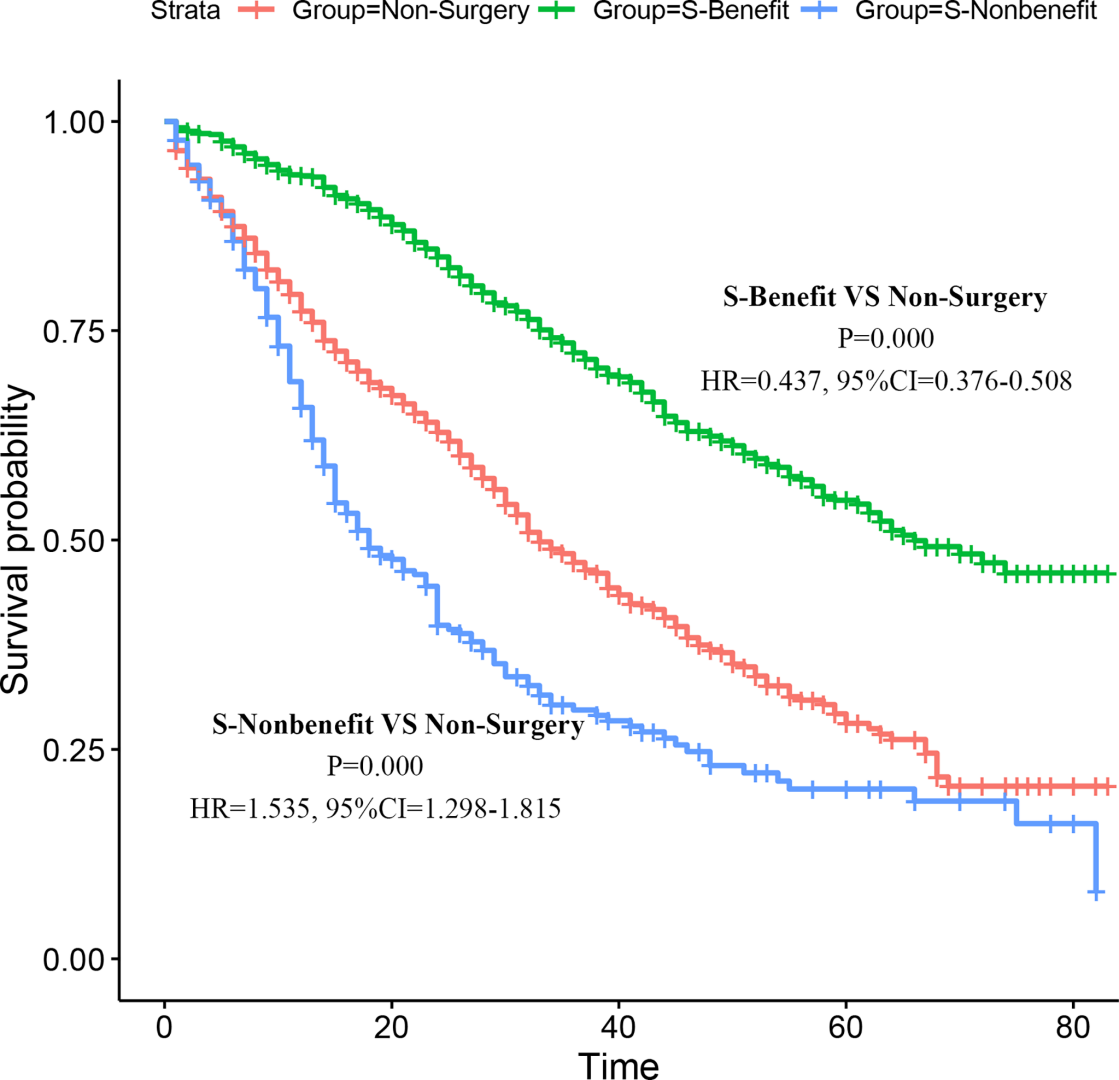
Validation of the nomogram in the PSM cohort. Kaplan–Meier (K-M) survival analysis of the patients in the S-Benefit, S-Nonbenefit, and Non-Surgery groups.

Moreover, to further verify the applicability of the model in the absence of prospective research data, we went back to the PSM cohort for application validation. As described before, 728 patients with stage IV BIDC were assigned to the Surgery-Benefit (S-Benefit) group, 300 patients were assigned to the Surgery-Nonbenefit (S-Nonbenefit) group, and the remaining 1,028 were in the Non-Surgery group. Subsequently, we generated the K-M survival curves with the log-rank test to verify the discrimination ability of the screening nomogram **(** **Figure 7** **)**. The results showed that patients in the S-Benefit group had a higher survival advantage than patients in the Non-surgery group (HR = 0.437, 95% CI, 0.376–0.508, p < 0.001), whereas patients in the S-Nonbenefit group presented poorer CSS than patients in the Non-Surgery group (HR = 1.535, 95% CI, 1.298–1.815, p < 0.001). These data further confirmed our hypothesis that not all stage IV BIDC patients would benefit from primary tumor surgery, and some patients may even have shorter postoperative survival time.

### Clinical Application and Significance

To apply the nomogram, we first draw a vertical line up from the corresponding point of each variable to obtain the corresponding score and then add up the scores of each variable and draw a vertical line down from the total score row to get the probability of benefit from primary tumor resection **(** **Figure 3** **)**. For example, if a widow with stage IV BIDC presents liver metastases, T4, histological grade IV, and molecular subtype HR-/HER2+, she will have a total score of 246, corresponding to an OR value of 0.726 (<1), indicating that she may not obtain a survival advantage from primary tumor resection. The novel nomogram is expected to be an effective screening tool for quantifying surgical benefit in female patients with stage IV BIDC, which may help oncologists make clinical decisions.

## Discussion

Approximately 6%–10% of female patients were diagnosed with stage IV BC (18), and about 20%–30% of early-stage patients would develop distant metastasis (21). Stage IV BC occurs when the tumor metastasizes from the breast and axilla to distant sites, most often to the bone (48%), brain (15%), liver, and lungs (21–23). The role of primary site surgery in the treatment of stage IV BC is still controversial. First, our study showed that primary site surgery improved prognosis in female patients with stage IV BIDC. The second aim of this study was to develop a nomogram to select candidates for locoregional surgery in female patients with stage IV BIDC and verify our hypothesis that not all female patients with stage IV BIDC are suitable for primary tumor resection.

The conventional view is that resection of the primary tumor in patients with stage IV BC is not associated with prolonged survival (except in patients with bone disease), and surgery may be only considered for certain patients whose systemic disease is under control, mainly to improve quality of life (QoL) (24–26). However, several recent retrospective population-based studies have shown that primary tumor resection has a positive effect on survival in patients with stage IV BC (18, 27–29). The earliest and largest relevant retrospective study was implemented by Khan and colleagues of the National Cancer Database (NCDB) from 1990 to 1993, in which they included 16,023 patients with stage IV BC. Of the patients, 42.8% did not undergo primary site surgery and 57.2% did (including partial and total mastectomy). The observed 3-year OS rate was 24.9%, including 31.8% for total mastectomy, 27.7% for partial mastectomy, and 17.3% for non-surgery (17). Additionally, a recent retrospective cohort study included 21,372 female patients diagnosed with stage IV BC between 1988 and 2011 and reached the similar result. Patients who underwent surgery had a longer median OS time than those who did not (28 months vs. 19 months) (18). However, the conclusions of these trials may be influenced by selection bias, as patients undergoing surgery tended to be younger or have only one metastatic lesion. Therefore, to address this problem, the propensity score matching (PSM) method was applied in our study to balance the clinical and pathological characteristics in the surgery and non-surgery groups and reduce selection bias. The results of these retrospective studies provided consistent and strong evidence for our finding that patients with stage IV BC who underwent primary tumor resection achieved better survival (30, 31). In addition, as with previous findings, we found that locoregional surgery was apparently an independent protective factor for survival (HR = 0.673, p < 0.001) by multivariate Cox regression analysis. Previous studies indicated that marginal status, systemic therapy, HER2 expression, number of metastatic sites, and type of metastatic disease were independent factors affecting the prognosis of BC patients (32–34). In contrast to these studies, we integrated different variables and further found that older age, higher T stage, higher histologic grade, distant metastases other than bone metastases (especially brain metastases, HR = 2.037), triple-negative breast cancer, and non-married status were associated with poor survival outcomes. Furthermore, in future work, we should compare the risk factors between the surgery group and all patients. The factors that lead to poor prognosis in the surgery group and do not affect the prognosis of all patients can be regarded as the signal of not accepting surgery, which is very valuable. Although retrospective studies have shown that primary tumor resection improves survival in patients with stage IV BC, there is insufficient evidence for prospective studies. The results of two international prospective studies (NCT00193778 trial and MF07-01 trial) on the role of locoregional surgery in the survival of patients with stage IV BC were inconsistent, primarily related to differences in postoperative systemic treatment. Thus, there is an urgent need for larger, multicenter prospective studies.

Although surgical interventions have shown better survival outcomes, whether all patients should undergo locoregional surgery needs further discussion, especially in patients with poor surgical tolerance. The unplanned subgroup of the MF07-01 trial found a survival benefit of breast surgery in young patients (<55 years) with bone metastases only and positive ER/PR status (35). In addition, another retrospective study indicated that stage IV BC patients with only bone metastases had prolonged postoperative survival (30). Existing studies lack a screening model to identify candidates for primary site surgery, so our study developed a nomogram to quantify the probability of surgical benefit. Our research suggested that seven independent indicators are associated with whether patients benefit from locoregional surgery, including histological grade, T stage, molecular subtype, lung metastasis, liver metastasis, brain metastasis, and marital status. Molecular subtype was most strongly associated with the probability of surgical benefit, followed by the histologic grade and brain metastasis. First, patients with stage IV BIDC of the molecular subtype HER2-/ER- were least likely to benefit from locoregional surgery. The reason may be that TNBC is the most aggressive group of BC, with poor prognosis and high recurrence rate. In addition, previous studies have reported that basal BC is prone to visceral metastases, especially brain and lung (36–38). Besides, our study suggested that patients with higher histologic grade of tumor were less likely to benefit from surgery. This can be explained by the fact that histologic grade is widely recognized as an important prognostic factor and that tumors with higher histologic grade are more prone to metastasize and relapse. We found that patients with brain metastases were less likely to benefit from surgery, possibly due to rapid disease progression, poor quality of life, and high mortality (21). In addition, Li et al. confirmed that patients only with brain metastases had a lower survival rate than patients with multisite metastases (excluding brain metastases) (39). Moreover, advanced T stage was found to have a negative effect on surgical benefit, which is consistent with the previous conclusion that advanced tumors suitable for surgical treatment should be small. It may be that the larger the tumor, the more likely it is to invade the chest wall, the more difficult it is to ensure a negative surgical margin (40). Hence, our screening nomogram incorporating these predictors may be useful for quantifying the probability of surgical benefit, providing guidance for further personalized clinical management.

To our knowledge, this is the first screening nomogram to quantify the probability of surgical benefit in female patients with stage IV BIDC and identify surgical candidates clinically. However, our study has some shortcomings. First, the information in the SEER database is not complete, such as surgical method, endocrine or targeted therapy status, and general health status of patients. Second, although the screening nomogram was established in the training set, and verified in the testing set, entire cohort, and PSM cohort, prospective studies are still needed. Third, as a retrospective study, only patients with stage IV BIDC at initial diagnosis were included, and metastatic disease that occurred in the latter stage cannot be identified, which may reduce some clinical guidance value.

## Conclusion

Our study suggested that primary tumor resection improved the survival of female patients with stage IV BIDC and developed a nomogram to quantify the probability of surgical benefit to help identify surgical candidates, which may help oncologists make clinical decisions.

## Data Availability Statement

Publicly available datasets were analyzed in this study. These data can be found here: https://seer.cancer.gov/.

## Author Contributions

ZQW, BC, and XXD conceived of and designed the study. JYC, ZXW, and HYG performed the literature search. BC and YW generated the figures and tables. ZQW and BC analyzed the data and wrote the manuscript. XXD critically reviewed the manuscript and supervised the research. All authors contributed to the article and approved the submitted version.

## Conflict of Interest

The authors declare that the research was conducted in the absence of any commercial or financial relationships that could be construed as a potential conflict of interest.

## Publisher’s Note

All claims expressed in this article are solely those of the authors and do not necessarily represent those of their affiliated organizations, or those of the publisher, the editors and the reviewers. Any product that may be evaluated in this article, or claim that may be made by its manufacturer, is not guaranteed or endorsed by the publisher.

## Supplementary Material

Supplementary Figure 1 The receiver operating characteristic curve **(A)**, calibration curve **(B)**, and decision curve analysis **(C)** of the screening nomogram in the entire cohort.

Supplementary Table 1 Comparison of clinical characteristics for the surgery group between before and after PSM.

Supplementary Table 2 Comparison of clinical characteristics for the non-surgery group between before and after PSM.

Supplementary Table 3 Comparison of median survival time and survival rate between the surgery and non-surgery groups.

Supplementary Table 4 The data of stage IV BIDC patients in the surgery and non-surgery groups before PSM.

Supplementary Table 5 The data of stage IV BIDC patients in the surgery and non-surgery groups after PSM.
