# Supplementary figures and images for: A Novel Nomogram Model to Identify Candidates and Predict the Possibility of Benefit From Primary Tumor Resection Among Female Patients With Metastatic Infiltrating Duct Carcinoma of the Breast: A Large Cohort Study
Source: Front Oncol. 2022 Feb 14;12:798016. doi: 10.3389/fonc.2022.798016 (PMC8883058; doi:10.3389/fonc.2022.798016)

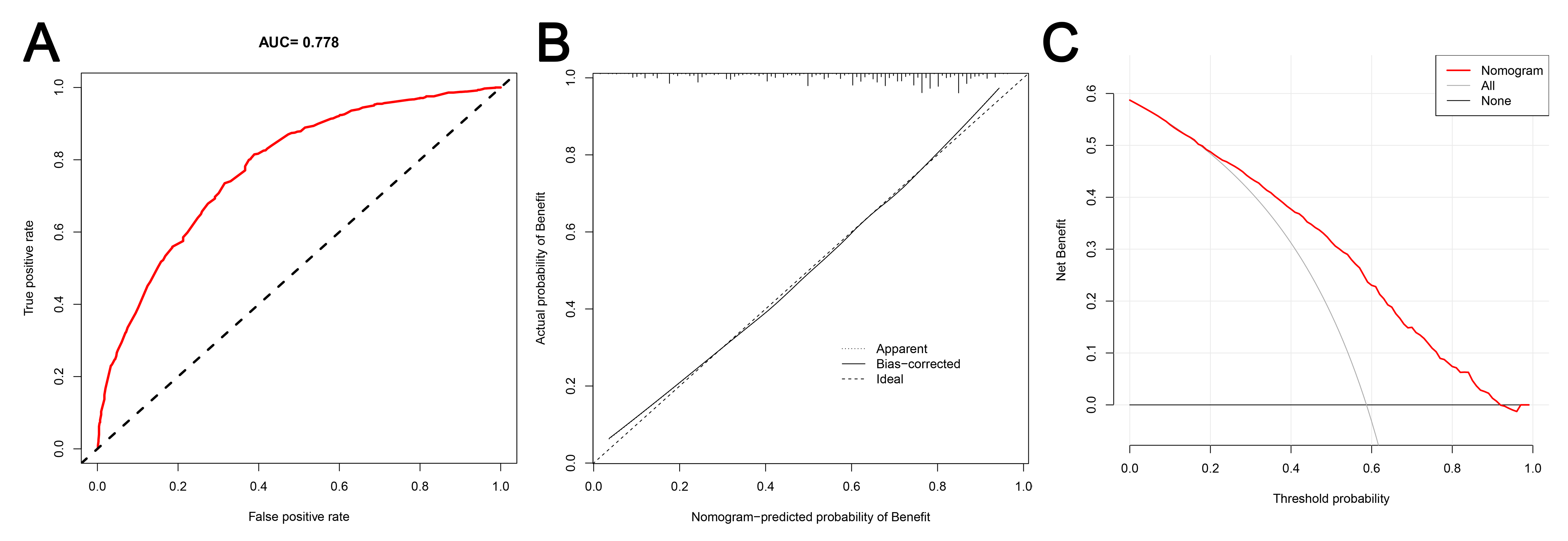

Supplement: Supplementary Figure 1 — The receiver operating characteristic curve (A), calibration curve (B), and decision curve analysis (C) of the screening nomogram in the entire cohort. [file Image_1.tif]
