# Supplementary material for: A Novel Nomogram Model to Identify Candidates and Predict the Possibility of Benefit From Primary Tumor Resection Among Female Patients With Metastatic Infiltrating Duct Carcinoma of the Breast: A Large Cohort Study
Source: Front Oncol. 2022 Feb 14;12:798016. doi: 10.3389/fonc.2022.798016 (PMC8883058; doi:10.3389/fonc.2022.798016)
Supplement: Supplementary Table 1 — Comparison of clinical characteristics for the surgery group between before and after PSM. [file Table_1.docx]

| **Table 1**. Comparison of clinical characteristics for the surgery group before and after PSM | | | | |
| --- | --- | --- | --- | --- |
|  | Before PSM (n=2375) | After PSM (n=1028) | X^2^ | P |
| Age, years |  |  | 5.282 | 0.071 |
| ≤40 | 333 | 126 |  |  |
| 41-60 | 1172 | 485 |  |  |
| ≥61 | 870 | 417 |  |  |
| Race |  |  | 1.045 | 0.593 |
| Black | 430 | 173 |  |  |
| Other | 217 | 90 |  |  |
| White | 1728 | 765 |  |  |
| Primary site |  |  | 3.958 | 0.555 |
| Central portion | 152 | 75 |  |  |
| Upper inner | 198 | 75 |  |  |
| Lower inner | 120 | 52 |  |  |
| Upper outer | 700 | 298 |  |  |
| Lower outer | 162 | 58 |  |  |
| Others | 1043 | 470 |  |  |
| Laterality |  |  | 0.132 | 0.717 |
| Left | 1213 | 532 |  |  |
| Right | 1162 | 496 |  |  |
| Grade |  |  | 23.517 | 0.000 |
| I | 113 | 59 |  |  |
| II | 734 | 398 |  |  |
| III+IV | 1528 | 571 |  |  |
| T |  |  | 1.530 | 0.675 |
| T1 | 286 | 130 |  |  |
| T2 | 981 | 414 |  |  |
| T3 | 429 | 174 |  |  |
| T4 | 679 | 310 |  |  |
| N |  |  | 6.454 | 0.011 |
| N0 | 369 | 196 |  |  |
| N1-3 | 2006 | 832 |  |  |
| Radiotherapy |  |  | 87.150 | 0.000 |
| No | 1168 | 684 |  |  |
| Yes | 1207 | 344 |  |  |
| Chemotherapy |  |  | 24.797 | 0.000 |
| No | 549 | 321 |  |  |
| Yes | 1826 | 707 |  |  |
| Bone metastasis |  |  | 22.775 | 0.000 |
| No | 1059 | 368 |  |  |
| Yes | 1316 | 660 |  |  |
| Brain metastasis |  |  | 2.289 | 0.130 |
| No | 2284 | 977 |  |  |
| Yes | 91 | 51 |  |  |
| Liver metastasis |  |  | 3.949 | 0.047 |
| No | 1853 | 770 |  |  |
| Yes | 522 | 258 |  |  |
| Lung metastasis |  |  | 1.441 | 0.230 |
| No | 1804 | 761 |  |  |
| Yes | 571 | 267 |  |  |
| Breast subtype |  |  | 14.019 | 0.003 |
| HR-/HER2- | 447 | 141 |  |  |
| HR-/HER2+ | 269 | 112 |  |  |
| HR+/HER2- | 1199 | 561 |  |  |
| HR+/HER2+ | 460 | 214 |  |  |
| Tumor size |  |  | 0.785 | 0.675 |
| ≤20 | 331 | 155 |  |  |
| 21-50 | 1174 | 504 |  |  |
| ≥51 | 870 | 369 |  |  |
| Insurance status |  |  | 0.179 | 0.672 |
| Uninsured | 71 | 28 |  |  |
| Insured | 2304 | 1000 |  |  |
| Marital status |  |  | 3.617 | 0.306 |
| Married | 1258 | 538 |  |  |
| Discovered | 349 | 135 |  |  |
| Single | 534 | 235 |  |  |
| Widowed | 234 | 120 |  |  |

IDC: Infiltrating duct carcinoma; PSM: propensity score matching.
