# Supplementary material for: A Novel Nomogram Model to Identify Candidates and Predict the Possibility of Benefit From Primary Tumor Resection Among Female Patients With Metastatic Infiltrating Duct Carcinoma of the Breast: A Large Cohort Study
Source: Front Oncol. 2022 Feb 14;12:798016. doi: 10.3389/fonc.2022.798016 (PMC8883058; doi:10.3389/fonc.2022.798016)
Supplement: Supplementary Table 2 — Comparison of clinical characteristics for the non-surgery group between before and after PSM. [file Table_2.docx]

| **Table 2**. Comparison of clinical characteristics for the non-surgery group before and after PSM | | | | |
| --- | --- | --- | --- | --- |
|  | Before PSM (n=3100) | After PSM (n=1028) | X^2^ | P |
| Age, years |  |  | 1.970 | 0.373 |
| ≤40 | 310 | 116 |  |  |
| 41-60 | 1464 | 492 |  |  |
| ≥61 | 1326 | 420 |  |  |
| Race |  |  | 1.745 | 0.418 |
| Black | 565 | 171 |  |  |
| Other | 271 | 85 |  |  |
| White | 2264 | 772 |  |  |
| Primary site |  |  | 3.937 | 0.559 |
| Central portion | 198 | 67 |  |  |
| Upper inner | 239 | 81 |  |  |
| Lower inner | 133 | 39 |  |  |
| Upper outer | 848 | 311 |  |  |
| Lower outer | 199 | 65 |  |  |
| Others | 1483 | 465 |  |  |
| Laterality |  |  | 0.037 | 0.846 |
| Left | 1600 | 527 |  |  |
| Right | 1500 | 501 |  |  |
| Grade |  |  | 9.858 | 0.007 |
| I | 186 | 40 |  |  |
| II | 1337 | 421 |  |  |
| III+IV | 1577 | 567 |  |  |
| T |  |  | 8.487 | 0.037 |
| T1 | 392 | 130 |  |  |
| T2 | 1066 | 392 |  |  |
| T3 | 606 | 210 |  |  |
| T4 | 1036 | 296 |  |  |
| N |  |  | 4.797 | 0.029 |
| N0 | 701 | 199 |  |  |
| N1-3 | 2399 | 829 |  |  |
| Radiotherapy |  |  | 6.180 | 0.013 |
| No | 2132 | 664 |  |  |
| Yes | 968 | 364 |  |  |
| Chemotherapy |  |  | 12.274 | 0.000 |
| No | 1171 | 326 |  |  |
| Yes | 1929 | 702 |  |  |
| Bone metastasis |  |  | 8.061 | 0.005 |
| No | 953 | 365 |  |  |
| Yes | 2147 | 663 |  |  |
| Brain metastasis |  |  | 15.563 | 0.000 |
| No | 2828 | 977 |  |  |
| Yes | 272 | 51 |  |  |
| Liver metastasis |  |  | 7.631 | 0.006 |
| No | 2145 | 758 |  |  |
| Yes | 955 | 270 |  |  |
| Lung metastasis |  |  | 27.610 | 0.000 |
| No | 1978 | 748 |  |  |
| Yes | 1122 | 280 |  |  |
| Breast subtype |  |  | 2.058 | 0.560 |
| HR-/HER2- | 418 | 154 |  |  |
| HR-/HER2+ | 302 | 93 |  |  |
| HR+/HER2- | 1757 | 585 |  |  |
| HR+/HER2+ | 623 | 196 |  |  |
| Tumor size |  |  | 0.829 | 0.661 |
| ≤20 | 483 | 155 |  |  |
| 21-50 | 1436 | 493 |  |  |
| ≥51 | 1181 | 380 |  |  |
| Insurance status |  |  | 4.077 | 0.043 |
| Uninsured | 156 | 36 |  |  |
| Insured | 2944 | 992 |  |  |
| Marital status |  |  | 3.233 | 0.357 |
| Married | 1460 | 517 |  |  |
| Discovered | 474 | 147 |  |  |
| Single | 821 | 259 |  |  |
| Widowed | 345 | 105 |  |  |

IDC: Infiltrating duct carcinoma; PSM: propensity score matching.
