# Supplementary material for: A Novel Nomogram Model to Identify Candidates and Predict the Possibility of Benefit From Primary Tumor Resection Among Female Patients With Metastatic Infiltrating Duct Carcinoma of the Breast: A Large Cohort Study
Source: Front Oncol. 2022 Feb 14;12:798016. doi: 10.3389/fonc.2022.798016 (PMC8883058; doi:10.3389/fonc.2022.798016)
Supplement: Supplementary Table 3 — Comparison of median survival time and survival rate between the surgery and non-surgery groups. [file Table_3.docx]

**Table 3**. Comparison of median survival time and survival rate between the surgery and non-surgery groups

| Before PSM | | After PSM |
| --- | --- | --- |
|  | Surgery Vs non-Surgery (95% CI) | Surgery Vs non-Surgery (95%CI) |
| Median OS | 49 (45.59-52.41) Vs 30 (28.35-31.65) | 46 (41.71-50.29) Vs 32 (29.23-34.77) |
| Median CSS | 55 (50.43-59.57) Vs 32 (30.11-33.89) | 53 (46.84-59.16) Vs 33 (30.05-35.95) |
| 1 year OS rate | 0.868 (0.854-0.882) Vs 0.727 (0.712-0.743) | 0.852 (0.831-0.874) Vs 0.761 (0.736-0.788) |
| 1 year CSS rate | 0.878 (0.865-0.892) Vs 0.745 (0.729-0.761) | 0.863 (0.842-0.885) Vs 0.773 (0.748-0.800) |
| 2 years OS rate | 0.713 (0.694-0.732) Vs 0.564 (0.546-0.583) | 0.706 (0.678-0.735) Vs 0.611 (0.581-0.643) |
| 2 years CSS rate | 0.730 (0.711-0.749) Vs 0.588 (0.570-0.607) | 0.725 (0.697-0.753) Vs 0.629 (0.598-0.661) |
| 3 years OS rate | 0.597 (0.576-0.619) Vs 0.430 (0.411-0.451) | 0.592 (0.560-0.625) Vs 0.454 (0.421-0.490) |
| 3 years CSS rate | 0.616 (0.595-0.638) Vs 0.455 (0.435-0.476) | 0.614 (0.583-0.648) Vs 0.473 (0.439-0.510) |
| 4 years OS rate | 0.503 (0.480-0.527) Vs 0.322 (0.302-0.344) | 0.486 (0.451-0.523) Vs 0.349 (0.313-0.389) |
| 4 years CSS rate | 0.533 (0.510-0.557) Vs 0.347 (0.326-0.370) | 0.523 (0.488-0.561) Vs 0.369 (0.332-0.410) |
| 5 years OS rate | 0.430 (0.405-0.456) Vs 0.242 (0.219-0.266) | 0.416 (0.379-0.456) Vs 0.258 (0.218-0.305) |
| 5 years CSS rate | 0.466 (0.441-0.493) Vs 0.265 (0.241-0.291) | 0.459 (0.422-0.501) Vs 0.281 (0.240-0.330) |

PSM: Propensity score match; HR: Hazard rate; OS: Overall survival; CSS: Cancer-specific survival; CI: Confidence interval.
